# Effect of the switch status of *Helicobacter pylori* outer inflammatory protein A on gastric diseases

**DOI:** 10.1186/s13568-023-01621-z

**Published:** 2023-10-10

**Authors:** Sinem Oktem-Okullu, Tayyip Karaman, Sümeyye Akcelik-Deveci, Emel Timucin, Osman Ugur Sezerman, Nesteren Mansur-Ozen, Yaren Buyukcolak, Arzu Tiftikci

**Affiliations:** 1 Department of Medical Microbiology, School of Medicine, Acibadem Mehmet Ali Aydinlar University, Atasehir, Istanbul, 34752 Turkey; 2 Department of Medical Biotechnology, Institute of Health and Science, Acibadem Mehmet Ali Aydinlar University, Atasehir, Istanbul, 34752 Turkey; 3 Department of Biostatistics and Medical Informatics, School of Medicine, Acibadem Mehmet Ali Aydinlar University, Atasehir, Istanbul, 34752 Turkey; 4 Department of Internal Medicine, School of Medicine, Acibadem Mehmet Ali Aydinlar University, Atasehir, Istanbul, 34752 Turkey

**Keywords:** *H. pylori*, OipA (outer inflammatory protein A), Gene switch status, Gastric diseases

## Abstract

*Helicobacter pylori* OipA (Outer Inflammatory Protein A) is an outer membrane protein that takes role in the adherence and colonization to the stomach. *oipA* gene expression is regulated by the slipped-strand mispairing mechanism through a hypermutable CT dinucleotide repeat motif in the 5΄ region. Alterations in the CT number repeats cause frame-shift mutations to result in phase variation of *oipA* expression. While a functional “On” status has been recognized as a risk factor for peptic ulcer diseases and gastric cancer in many studies, some controversial findings still exist. To this end, this study compiled the sequence data of *oipA* from 10 different studies between 2000–2019 and 50 *oipA* DNA sequences from our own research that examined the relationship between the phase On/Off status of *oipA* and gastric diseases based on CT repeat number. Overall, we have reached 536 *oipA* DNA sequences from patients. This large collection of *oipA* sequences first clarified the absolute conservation of the peptide-pentamer of FWLHA for phase *‘’*On’’ status, suggesting this pentamer as a superior marker for the determination of *oipA* status than counting the number of CT repeats. Combining the sequence and patient data, we have re-analyzed the association between the ‘‘On’’ status of *oipA* and gastric diseases. Our results showed a strong association between *oipA* ‘‘On’’ status and gastric cancer supporting previous findings. We also investigated the AlphaFold2 computed structure of OipA that adopts a beta-barrel fold closely resembling to the autotransporter family of *H. pylori*. Altogether, this study confirms a strong association between *oipA* ‘‘On’’ statuses and severe gastrointestinal diseases like cancer and provides useful insights into the FWLHA pentamer as an indicator of “On” status of *oipA* putative autotransporter function rather than CT repeats number.

**Supplementary Information:**

The online version contains supplementary material available at 10.1186/s13568-023-01621-z.

## Introduction

*Helicobacter pylori* is a Gram-negative pathogenic bacterium that lives in harsh conditions of the human stomach and colonizes gastric epithelial cells. More than half of the human population in the world is infected by *H. pylori* which causes peptic ulcer diseases, gastritis, gastric erosion, intestinal metaplasia, and gastric cancer (Armitano et al. 2013; Chiarini et al. 2009; Sallas et al. 2019; Torres et al. 2014). Although the mechanism of pathogenesis by *H. pylori* has not been fully understood, most of its virulence factors are reported to play roles in such gastrointestinal diseases in humans (Casadevall and Pirofski 2009) such as CagA, VacA, DupA, BabA, SabA, AlpA/B, OipA (Chiarini et al. 2009; Conradi et al. 2012). The presence of virulence factors is linked to the severity of diseases caused by *H. pylori* and the expression of these genes at different levels resulting in diversity in terms of disease severity. (Markovska et al. 2011; Braga et al. 2019; Farzi et al. 2018; Sallas et al. 2019; Yamaoka et al. 2000).

OipA is an outer membrane protein encoded by the *oipA* gene that is found in *H. pylori*. The functionality of the gene is determined by its status as “On” and “Off” which is regulated by the slipped-strand mispairing (SSM) mechanism that alters the CT dinucleotide repeats in the 5’ of the gene corresponding to the signaling peptide (Braga et al. 2019; Casadevall and Pirofski 2009; Dossumbekova et al. 2006; Farzi et al. 2018). This mechanism provides genomic plasticity to the bacteria ensuring its survival in extreme conditions. While a certain number of CT repeats results in successful OipA production to contribute *H. pylori* colonization in gastric epithelial cells, some number of CT repeats may lead to an early stop codon switching the gene status to off.

The major role of OipA is to provide *H. pylori* to adhere to the surrounding gastric epithelial cells. While IL-8 production is primarily induced by the translocation of CagA through the type IV secretion system (Horridge et al. 2017; Odenbreit et al. 2000), OipA has also been shown to induce IL-8 production in neighbor epithelial cells and increase the risk of the development of gastric cancer (Horridge et al. 2017; Yamaoka et al. 2000, 2002). Although the role of *oipA* and its action mechanism with other virulence factors “On” gastric diseases has been clarified to some extent, as a phase variable virulence factor, associations of *oipA* switch status with *H. pylori*-related gastric diseases remains controversial. Also, the structure of OipA and the exact mechanism of switch status are still unclear.

In this study, we collected all of the published sequence and disease data and analyzed them to understand the relationship between the switch status of *oipA* with gastrointestinal diseases. Our collected data containing 716 cases from more than 10 studies enlisted the 5’ region of the *oipA* gene, its switch status, and clinical outcomes (Dossumbekova et al. 2006; Markovska et al. 2011; Sallas et al. 2019; Torres et al. 2014) (Braga et al. 2019; de Jonge et al. 2004; Farzi et al. 2018; Fasciana et al. 2015; Yamaoka et al. 2000, 2002). Our results showed a strong association between “On” status of *oipA* and some of the gastrointestinal diseases. Overall, this study shed light onto a novel motif as an indicator of the “On” status of *oipA* and the first computed model of the full-length OipA structure.

## Materials and methods

### Patients

We included 50 subjects infected with *H. pylori* strains: 22 with chronic gastritis, 2 with gastric cancer, 3 with intestinal metaplasia, and 23 with peptic ulcer diseases who underwent upper endoscopy for evaluation of dyspeptic symptoms to Acıbadem Maslak Hospital, Gastroenterology Department, Istanbul, Turkey. The study was approved by the Acıbadem Mehmet Ali Aydinlar University and Acıbadem Healthcare Institutions Medical Research Ethics Committee (ATADEK). Gastric tissue samples to be included in the study were obtained from volunteer patients who agreed to participate in the study by signing the informed consent.

The DNA was extracted using Quick-DNA/RNA kits (Zymo Research) according to the manufacturer’s recommendations. The DNA concentration was determined by spectrophotometry using NanoDrop 2000 (Thermo Scientific, Wilmington, NC) and stored at − 20 °C until use.

The 5′ signal region of the *oipA* gene, including the CT dinucleotide repeats, was amplified by polymerase chain reaction (PCR) assay using a primer pair 5′- GTTTTTGATGCATGGGATTT − 3′ as a forward and 5′- GTGCATCTCTTATGGCTTT − 3′ as a reverse.

The amplified PCR products were subjected to Sanger sequencing sequenced in MedSanTek Genomics laboratories in Turkey, and the data was blasted to the designed DNA sequence. Sanger sequenced data was analyzed by using the BioEdit sequence alignment editor program and the aligned fragments were blasted with designed DNA fragments sequenced as a query at the NCBI page.

DNA sequences of the signal peptides collected from the patients were deposited to GenBank with the ID number 2,721,150. All the DNA and protein sequences of the signal peptides collected in this study and from literature with different *oipA* states are available within Supplementary Information 1.

### Data collection

Signal peptide sequences of *oipA* were obtained from eleven different studies and from our own cohort of fifty patients, overall resulting in 716 *H. pylori* infected cases. The number of CT repeats was counted, and the locations of the repeats were identified relative to the start codon of *oipA*. Both DNA and protein sequences were classified according to the “On” and “Off” status of *oipA*. Our data also include the number of patients who suffered from gastrointestinal diseases and their OipA protein status. The frequency table of CT repeats in the “On” and “Off” states and the frequency table of 4-long motifs in both “On” and “Off” status was provided in the supplementary materials.

### Multiple sequence alignment analysis

DNA and protein sequences of the signal peptides collected from the patients with different *oipA* states were aligned by Clustal Omega (Madeira et al. 2022). Phylogenetic relationships between the sequences were identified by the neighbor-joining method using Clustal Omega.

### Statistical analysis

Associations of *oipA* statuses with CT repeat patterns, clinical outcomes and 4-long motifs were analyzed by Fisher exact tests. Statistical analysis was carried out by R version 4.2.2. (Team 2014).

### Prediction of full-length structures of OipA, SabA and BabA

AlphaFold2 (AF2) Colab script was used to predict the tertiary structure of *H. pylori* of OipA, SabA and BabA autotransporters (Mirdita et al. 2022). BOCTOPUS2 was used to predict the transmembrane topology of all AF2-predicted structures (Hayat et al. 2016). Structural alignment was carried out by the matchmaker function of ChimeraX (Meng et al. 2006; Pettersen et al. 2004, 2021).

## Results

### Analyzing the relationship between ***oipA*** gene status and gastrointestinal diseases

**Fig. 1:**
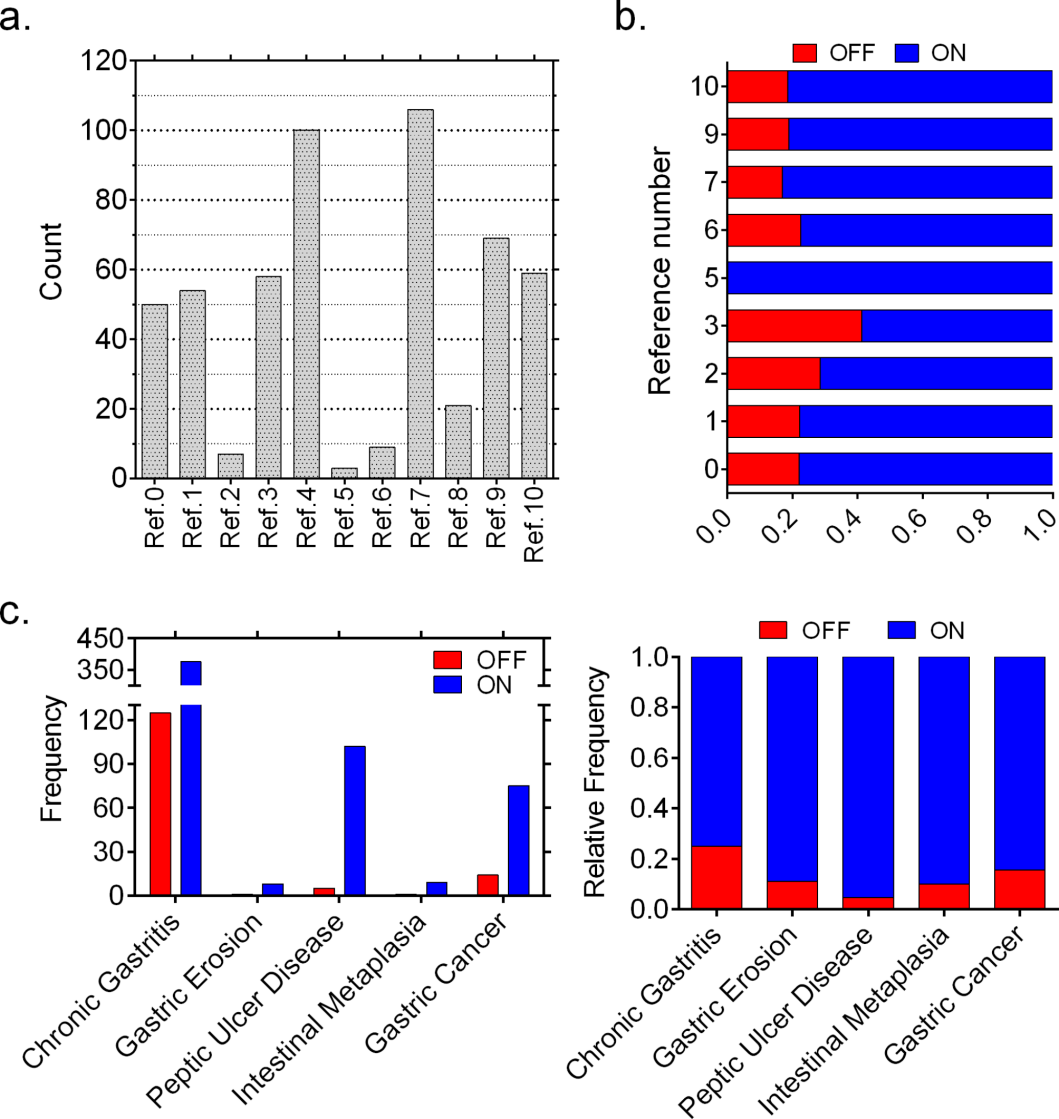
Functional status of *oipA* in gastrointestinal disease patients. (**a**) shows the number of *oipA* sequences in each study, (**b**) shows distribution of *oipA* On/Off status in the selected studies. (**c**) shows the percent distribution of *oipA* On/Off statuses of the studies from which sequences were collected. Reference number 0 corresponds to this study which contributed about 50 different *oipA* sequences

Including our cohort of fifty patients, we have collected the data of ten different studies, resulting in 716 cases that were associated with one of the gastric diseases listed in Table 1. Five different gastric diseases, which are chronic gastritis (CG), gastric erosion (GE), peptic ulcer disease (PUD), intestinal metaplasia (IM), and gastric cancer (GC), were reported by these cases. Among these, the most prevalent disease is CG consisting of 501 patients while GE and IM cases were the least encountered ones in the collected *H. pylori* cohort (Table 1). We encountered that a large fraction of the gastric disease cohort showed *oipA* ‘’On’’ status (80%) (Braga et al. 2019; Farzi et al. 2018; Sallas et al. 2019; Yamaoka et al. 2000) and reported a significant association between having an On *oipA* and gastric diseases (p < 0.05) (Table 1). Figure 1a shows distribution of number of *oipA* sequences in the reference studies. Ref.0 represents the patient data obtained from this study. Most of the studies contributed to the total cohort with more than 50 cases (Fig. 1a). Figure 1b shows how the *oipA* On/Off sequences were distributed within these references. Most of the studies showed a larger fraction of *oipA* ‘’On’’ status rather than ‘’Off’’. We also analyzed how *oipA* On/Off statuses were distributed across gastric diseases (Fig. 1c). *OipA* ‘’On’’ sequences were consistently much higher than ‘’Off’’ sequence across all diseases. Relative frequencies showed that *oipA* ‘’On’’ sequences were mostly spotted in the chronic gastritis and gastric cancer cases while peptic ulcer disease had the most unbalanced distribution having much fewer ‘’Off’’ sequences (Fig. 1c). This analysis compiling one of the largest patient data with diverse gastric disease outcomes and *oipA* statuses showed that functional *oipA* of *H. pylori* affects the host’s medical condition (SI1). Patients that are infected by *H. pylori* containing a functional *oipA* were prone to more severe gastrointestinal diseases and cancer.

**Table 1:** Functional status of *oipA* in gastrointestinal disease patients

Clinical outcome	Off	On	Total	p-value	Ref. #
	n (%)	n (%)	n		
Chronic Gastritis	125 (0.25)	376 (0.75)	501	< 0.001	0,1,2,3,5,6,7,9,10
Gastric Cancer	14 (0.16)	75 (0.84)	89	< 0.001	0,1,5,6,10
Gastric Erosion	1 (0.11)	8 (0.89)	9	0.02	1,0
Intestinal Metaplasia	1 (0.1)	9 (0.9)	10	0.011	0,1
Peptic Ulcer Disease	5 (0.05)	102 (0.95)	107	< 0.001	0,1,2,5,9
**Total**	146 (0.2)	570 (0.8)	716	0.002	

### Sequence analysis of the signal peptide of OipA

*H. pylori* regulates the On/Off statuses of some virulence genes such as *oipA, babA* and *sabA* by the slipped-strand mispairing (SSM) DNA repair system, a process which occurs during the replication of DNA sequences with repetitive regions (Ansari and Yamaoka 2019; Torres-Cruz and van der Woude 2003). Changes in the number of repeats often result in frame-shift mutations leading to phase variation in the expression of such genes. Having a poly CT-repeat region in the 5’ region, the *oipA* gene replication is prone to the mutagenic SSM process. As a result, *oipA* On/Off status often is determined by examining the number of CT repeats.

**Fig. 2:**
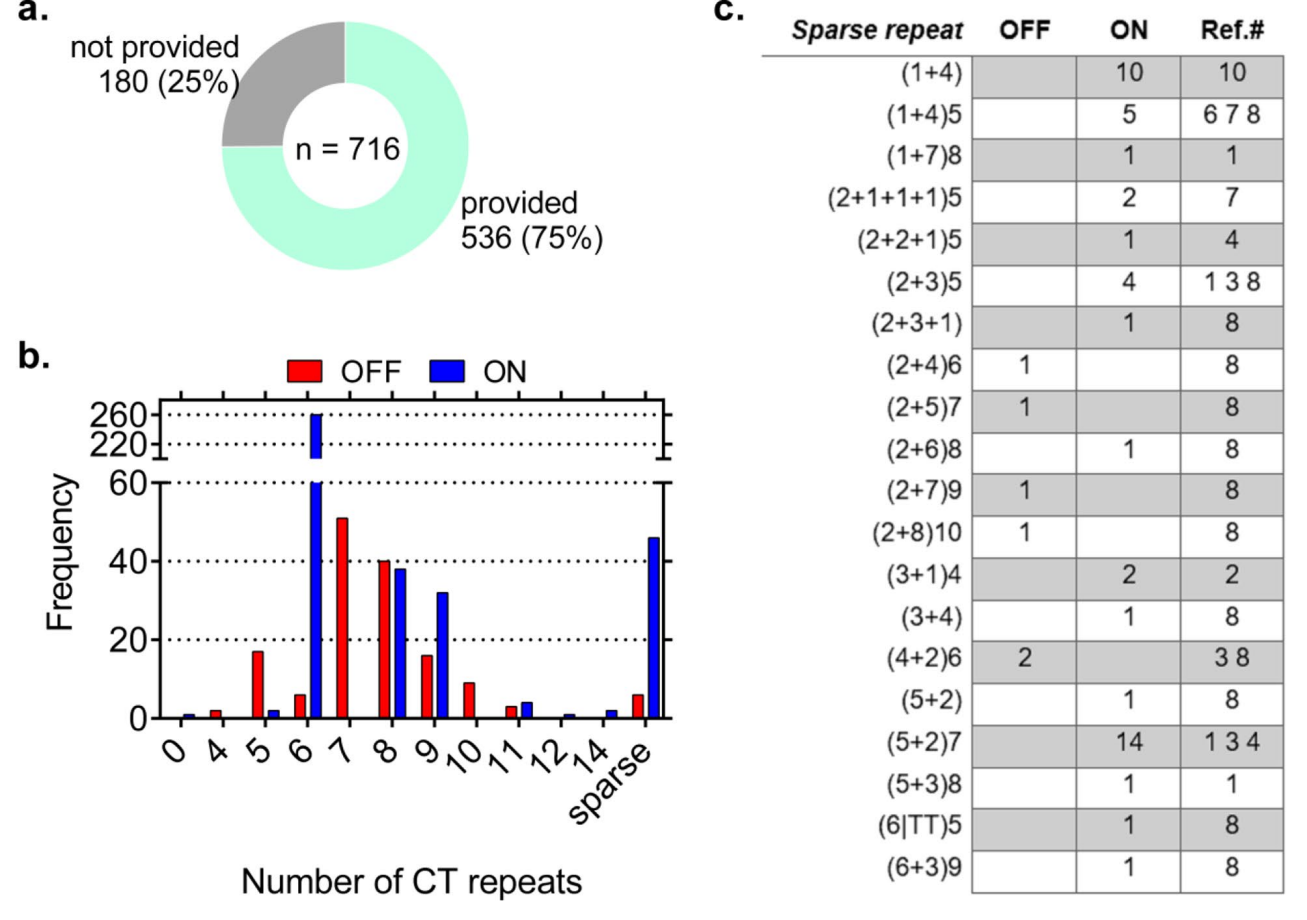
(**a**) Abundance of CT repeats and status. (**b**) Abundance of four nucleotide motifs with the status of *oipA*. (**c**) Sparse repeats based on *oipA* “Off” and “On” statuses

Although a total of 716 patient data were obtained from the literature, only 536 of them had OipA sequence information (Fig. 2a). In this subset, 386 cases had *oipA* genes in the ‘‘On’’ status and 150 of them were in the ‘‘Off’’ status. Inspecting these OipA sequences, we have identified 29 distinct patterns of CT dinucleotide repeats (Fig. 2b and c). Particularly 5 of the patterns including 5CT, 6CT, 8CT, 9CT, 11CT were observed both in the ‘’On’’ and ‘’Off’’ sequences of *oipA*. Although a significant association was reported between the functional status of *oipA* and the repeat patterns (p < 0.05), discrimination of *oipA* functional status based on dinucleotide counts or patterns was not possible. Thus, confirming a high variation in the number of CT dinucleotide repeats in the *oipA* sequences, we noted an absence of a pattern between the number of CT dinucleotide repeats and the functional status of *oipA*, reflecting the poor performance of dinucleotide repeat count in the determination of *oipA* On/Off status.

### Multiple sequence alignment of ***oipA*** sequences

**Fig. 3:**
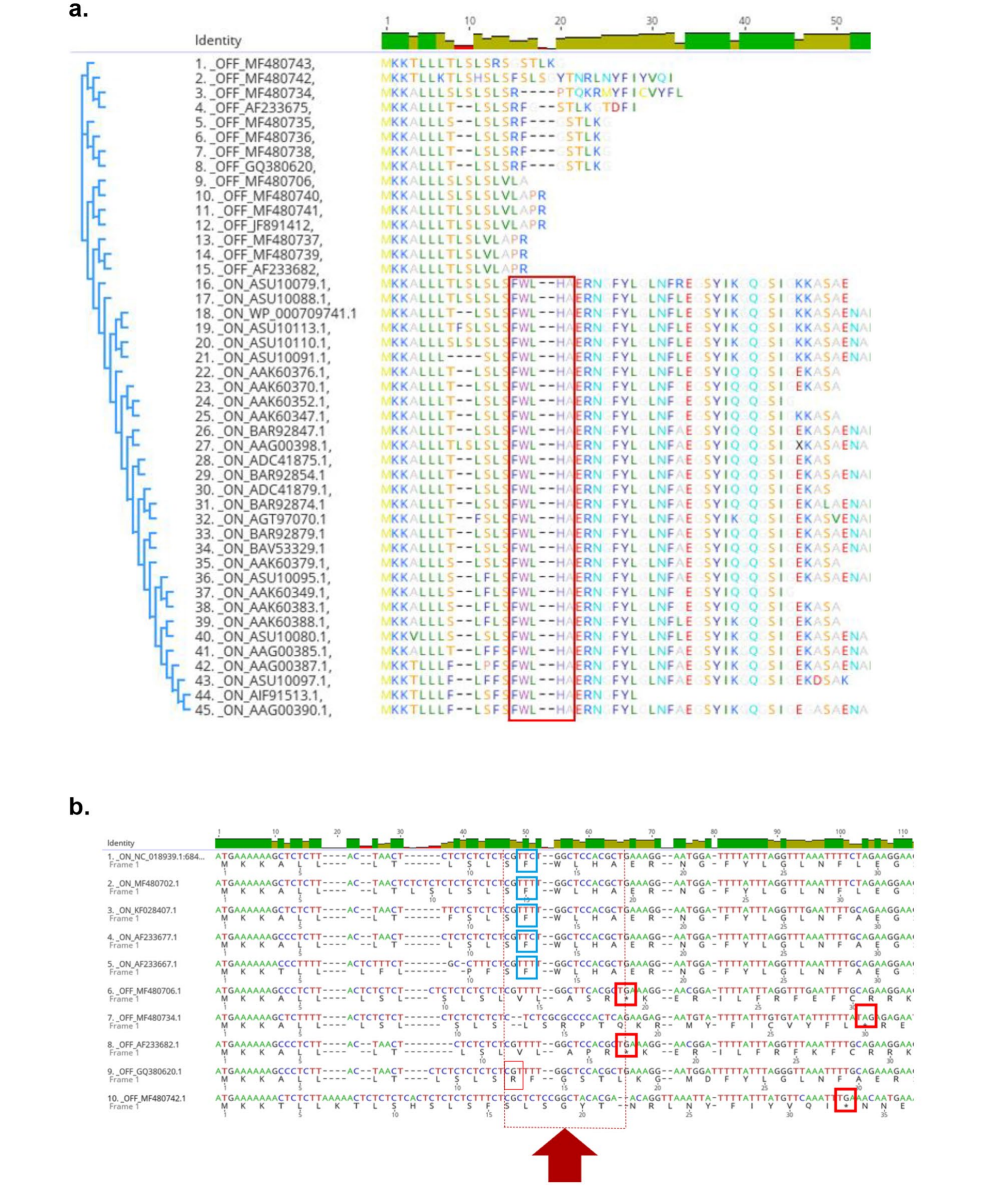
(**a**) Multiple alignment of “On” and “Off” status of OipA signaling peptide protein sequences. (**b**) Amino acid sequences of the OipA signal peptides that were collected from different studies were aligned by Clustal Omega

Amino acid sequences of the OipA signal peptides that were collected from different studies were aligned by Clustal Omega (Fig. 3a). Multiple sequence alignment of the collected sequences clarified that the ‘’FWLHA’’ pentamer is absolutely conserved in the *oipA* “On” strains of *H. pylori*. On the other hand, the signal peptide of OipA was annotated as off when a CT repeat pattern has caused a frameshift mutation, causing the loss of this pentamer in the amino acid sequence.

Since this motif is encountered in all *oipA* sequences with “On” status, it is proposed to play an essential role in the function of OipA. Among these five amino acids, the absence of tryptophan directly affects the signaling peptide to locate the OipA protein. It is a known fact that tryptophan’s bulky side chain found to be in interact with membrane interfaces for anchoring and hydrophobic mismatch response is regulated by tryptophan enabling the peptide to modulate structural perturbations (de Jesus and Allen 2013). All in all, the absence of tryptophan affects the functional status of OipA.

Figure 3: (a) Multiple alignment of “On” and ‘‘Off’’ status of OipA signaling peptide protein sequences. (b) Amino acid sequences of the OipA signal peptides that were collected from different studies were aligned by Clustal Omega.

Five representative “On” and five “Off” nucleotide sequences were also aligned to understand how CT dinucleotide repeats change the signaling peptide of the OipA protein. In all “on” sequences, CT repeats do not alter the last codons of signal peptide which are responsible for the transcription of the ‘’F-W-L-H-A’’ amino acid sequence. In “on” sequences, end of the signal sequence should follow, (TCG)-(TTT/TTC)-(TGG)-(CTC)-(CAC)-(GCT) series of codons. In contrast, CT repeats directly affect the formation of the F-W-L-H-A sequence at the end of the signaling peptide because of frameshift in “off” sequences. Altered codons generally cause the formation of a stop codon. In some cases, like 9th sequence (the one with the accession number of GQ380620) in Fig. 2b, stop codon is not observed but it still cannot function because of losing F-W-L-H-A sequence order. Generally, disarrangement starts with the phenylalanine (TTT/TTC) that comes right before tryptophan (TGG). As pointed in Fig. 3b, frameshift generally causes the formation of valine (GTT) or arginine (CGT) and it goes with a completely changed order of amino acids.

Multiple sequence alignments were used to construct a phylogenetic tree by using the Neighbor-Joining Tree method (SI2). Since “Off” status sequences generate a stop codon, we only checked the signaling peptide region during alignment, to compare the status difference properly. As a result of phylogenetic tree analysis, we concluded that “On” and “Off” strains are clustered separately. This indicates how disruption of the ‘’FWLHA’’ motif strongly affects the functional status of OipA.

### Multiple sequence alignment of SabA, BabA, and OipA

**Fig. 4:**
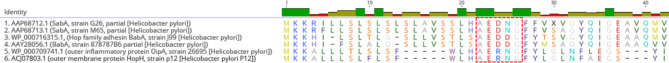
Multiple alignments of SabA, BabA and OipA protein sequences

SabA, BabA, and OipA belong to the *H. pylori* outer membrane protein family, so the possibility of their similarity in a protein sequence may give us significant information about the function of OipA. It’s a known fact that SabA and BabA have A↓EX[D/N]G motif in their protein sequence (“↓” represents the cleavage site of the protein) in a very conserved way (Coppens et al. 2018). As a result of multiple alignments, we concluded that OipA contains the same motif (Fig. 4) with SabA and BabA. The last three amino acid of the OipA signaling peptide, its cleavage site, and the beginning four amino acids of the remaining sequence fits exactly to the conserved A↓EX[D/N]G signature sequence of SabA and BabA. Despite the fact that supporting evidence is needed, this multiple alignment result indicates strong similarity of OipA with SabA and BabA autotransporters.

### Prediction of full-length structures

**Fig. 5:**
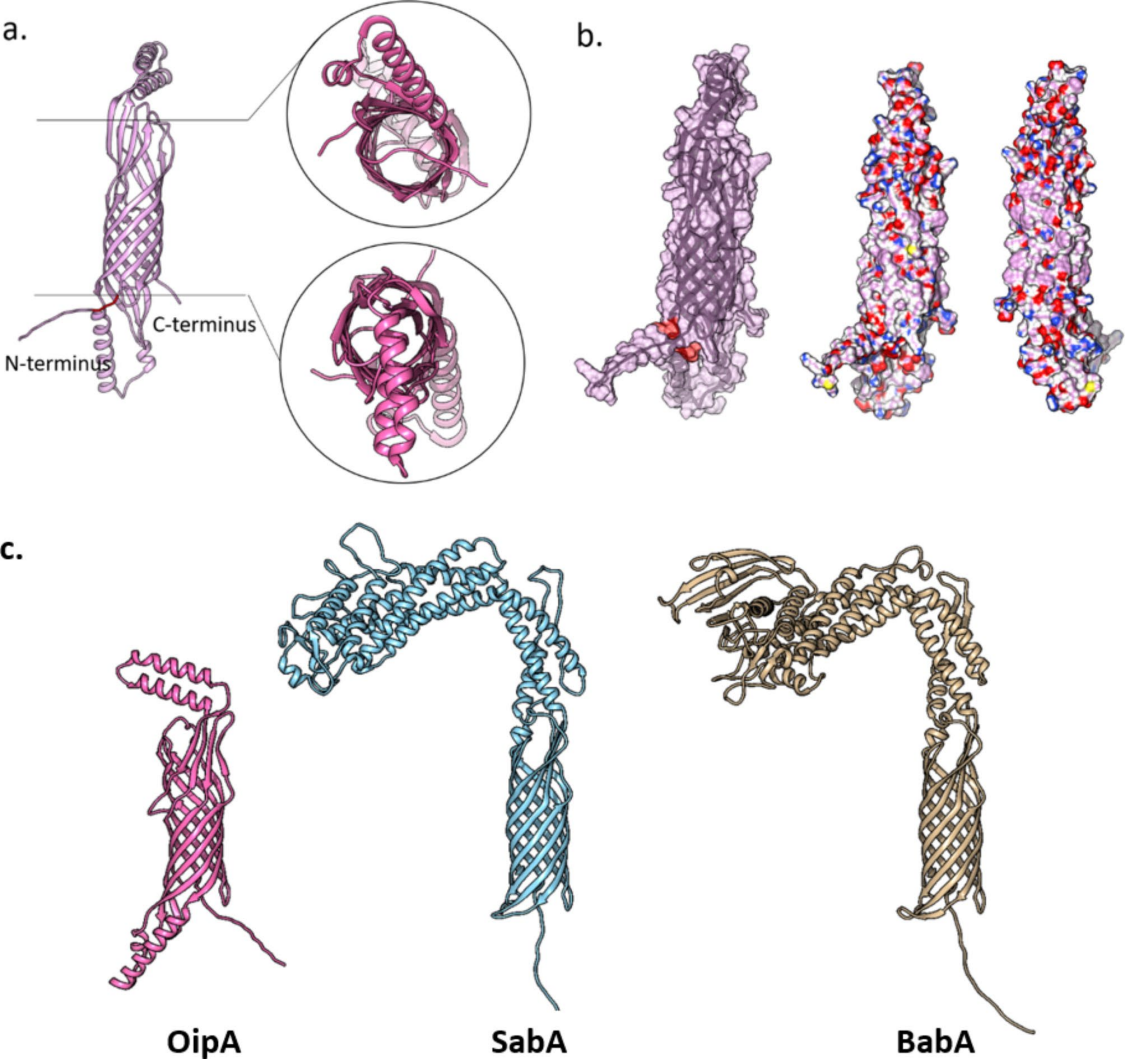
Predicted OipA structure at its full-length. (**a**) shows the backbone with the top and bottom cross-sections, the ‘’FWHLA’’ pentamer is colored red. (**b**) Surface view of the predicted structure with hydrophobic residues shown in pink. (**c**) Structural superimposition of OipA (pink), SabA (blue) and BabA (brown). Β-barrel domain of all three structure were aligned and Cα RMSD values for both superimpositions were as follows for the pruned atoms: OipA-SabA: 0.897 Å, OipA-BabA: 0.915 Å

The structure of OipA has been successfully predicted by AF2 (Fig. 5a). The resulting structure showed high prediction performance-based “on” per residue confidence score overall implying an accurate model. This structure of OipA, as the first ever model at the atomistic resolution, showed that the fold of OipA is a β-barrel with a likelihood of being an autotransporter (Coppens et al. 2018; Fan et al. 2016; Meuskens et al. 2019). Although the predicted structure does not show a passenger domain, which inserts itself into the barrel in autotransporters (Coppens et al. 2018; Meuskens et al. 2019), given high accuracy per residue and low predicted aligned error, the resulting AF2 structure underscored the plausibility of OipA of being an autotransporter.

Beta barrel structure of OipA provides a pore that enables a passage for specific substances. The such structure obtained from AF2 is found to be similar with the proteins that belong to Vf subclass of type V secretion systems which are also known as autotransporters. As a result of the structure similarity, we proposed that OipA may belong Vf class of Type V secretion system and it may provide translocation of a certain substance to induce IL-8 secretion (Coppens et al. 2018). The backbone structure and surface view of OipA was visualized and colored according to its hydrophobicity (Fig. 5). Middle part which is full of beta barrels of OipA is more hydrophobic comparing to other sites. Moreover, OipA does not have extracellular opening as a result of surface view (Fig. 5b).

### Structural alignment

The structure may give invaluable insights into the protein function. Thus, we also generated the structures of SabA and BabA which are known autotransporters and compared the structure of OipA with those. Particularly the β-barrel domains showed a high match suggesting a functional similarity in OipA with those of SabA and BabA (Fig. 5c). Given this alignment, we appraised that OipA as well could be an autotransporter.

### Prediction of transmembrane topology

The topology of OipA was analyzed by BOCTOPUS2 tool that enables to prediction topology of transmembrane beta-barrel proteins using the Hidden Markov Model. Besides that, proteins’ pore-facing, lipid-facing, inner-loop, and outer-loop regions can be predicted as a result of the Support Vector Machine algorithm. Six transmembrane regions were found “On” *oipA* sequence (*Helicobacter pylori* 26,695 strain -on status-) via Hidden Markov Model. Orientation information that contains pore-facing, lipid-facing, inner loop, and outer loop of the protein of interest are shown in Supplementary Data 3. After that, we checked other autotransporters SabA and BabA to analyze the similarity of OipA with other autotransporters that belong to *H. pylori*. We discovered very similar per-residue orientation prediction for OipA and other ATs. Orientation of proteins that are almost last 200 amino acids is showing that, OipA is most likely an autotransporter based “On” similarity in topology with other ATs of *H. pylori* SabA and BabA. Especially lipid-facing and pore-facing regions are quite alike.

## Discussion

In order to survive in the stomach’s acidic environment, alter the immune system, and damage the gastric epithelium, *H. pylori* secretes different virulence factors in the form of proteins. Virulence factors of *H. pylori* have a crucial role in getting severe gastric diseases. As a result of virulence effects, the host becomes vulnerable to get peptic ulcers, gastritis, gastric erosion, intestinal metaplasia, or gastric cancer (Braga et al. 2019; Sallas et al. 2019; Yamaoka et al. 2000).

OipA (outer inflammatory protein A) is an important virulence factor that plays a role in increasing the secretion of interleukin-8 (IL-8), production of proinflammatory factors to cause neutrophil infiltration, aggravating the inflammation in the stomach, and interaction with host cell membrane for colonization. In the literature, there have been controversial studies about the correlation between the *oipA* “On” status and the severity of gastric diseases. Our study partly addresses this controversy by compiling the largest *H. pylori*-infected patient data. Overall, results that show a significant increase in the number of cancer patients infected by *H. pylori* with a functional OipA suggest *oipA* ‘‘On’’ status as a strong risk factor for *H. pylori*-induced gastric cancer. This association could be related to the ability of OipA to increase formation of free radicals, which in turn leads to mutations in host cells (Bartpho et al. 2020) and to enhance neutrophil activity and IL-8 release (Bartpho et al. 2020; Torres et al. 2014; Yamaoka et al. 2000). Furthermore, *oipA* ‘‘On’’ status has been linked to DNA damage-induced genotoxicity and abnormal gene methylation in *H. pylori*-related gastric carcinogenesis (Vahidi et al. 2022). Altogether, our analysis of the most comprehensive meta-data supported with the patient data consolidated the association between *oipA* ‘‘On’’ status and gastric cancer reflecting the potential use of the status of *oipA* as a prognostic marker *H. pylori* infection.

The functional status of *oipA* is regulated by phase variation that occurs by the addition/deletion of CT repeat motifs located in the 5΄ regions of the gene (Vahidi et al. 2022; Yamaoka et al. 2002). Thus it is often determined based “on” the number of CT repeats in the DNA sequence, especially at the N-terminus where the signal peptide is located (Farzi et al. 2018; Vahidi et al. 2022). While accumulated evidence including our meta-analysis showed a strong association between oipA “On” status and gastric cancer, a high degree of variability in the number of CTs exists in the *H. pylori* strains isolated from patients interfering with determination of status of *oipA* (Fig. 1a). Nevertheless, number of CT repeats have still been used for determination of *oipA* functionality (Braga et al. 2019; de Jonge et al. 2004; Dossumbekova et al. 2006; Farzi et al. 2018; Fasciana et al. 2015; Markovska et al. 2011; Sallas et al. 2019; Torres et al. 2014; Yamaoka et al. 2000, 2002). DNA sequences analyzed in this study containing the entire sequence corresponding to signal peptide showed an absolute classification of *oipA* On/Off based “On” FWLHA pentamer. This motif resides at the C terminus of the signal peptide of OipA, which is cleaved at the alanine position of the pentamer (Coppens et al. 2018). Hence, we propose the ‘’FWLHA’’ protein motif that has been absolutely conserved in all *oipA* ‘‘On’’ sequences as a marker for the determination of *oipA* functional status rather than the number of CT repeats. To the best of our knowledge this is the first report “on” promoting the utilization of either FWLHA pentamer or TTC/TTT (F in codon) as markers for accurate determination of *oipA* switch status rather than the number of CT repeats.

Hops share a conserved transmembrane domain composed of non-continuous β-strands at both termini (Webb et al. 2017). As being a member of Hop family, OipA is an integral membrane protein with a large transmembrane domain, which interferes with its structural characterization. In line with this notion, other members of Hop family such as SabA-BabA do not have full length structures in PDB. Although, the extracellular domains of these proteins have been structurally characterized, structure of the transmembrane domain still remains unknown. Notably, AF2 computed models of OipA showed particularly high per residue confidence scores and low predicted aligned errors for the transmembrane domain, underlining an accurate prediction for the integral membrane part of OipA. This structure provided valuable insights into the function of OipA. First, it confirmed a non-continuous transmembrane domain which is a structural feature of hop family (Coppens et al. 2018). Furthermore, predictions of SabA- BabA structures showed a perfectly aligned transmembrane domain for all three hop members (Fig. 5c). Given this match in the transmembrane domains OipA is likely to share functional similarity with SabA-BabA. As such, OipA could act as an autotransporter, which is mostly found in Gram-negative bacteria and frequently linked to virulence processes such as adhesion. Along with the structural similarity between OipA and Saba-Baba, we also note that the A↓EX[D/N]G motif corresponding to the cleavage site of the signal peptide is also conserved in all three proteins (Fig. 4) taken together with the high sequence and structural similarities with other known autotransporters, namely SabA and BabA, we surmise that OipA plays an important role as an autotransporter in bacterial adherence to the gastric mucosa to initiate the colonization process.

After years of research into the intricate interaction between *H. pylori* and its human host, numerous factors that influence the patient’s health have been uncovered. These factors include, but not limited to, the individual’s genetic predisposition, gene regulation, environmental conditions, and the diversity of virulence factors in the *H. pylori* strain (Cadamuro et al. 2014). This study, focusing a single virulence factor that could affect the severity of the *H. pylori* infection, would be limited to address different host-related factors that could also play critical role in *H. pylori* infection.

*Helicobacter pylori* OipA (Outer Inflammatory Protein A) play an important role in the bacterial adherence and colonization. Although many studies have reported that a functional “On” status of this gene as a risk factor for gastric diseases, most of them have utilized small sample sizes. To that end, this study used the largest dataset to investigate the relationship between the status of OipA status and having a gastric disease. Our findings contributed to the determination of the functional status of OipA from its signal sequence, proposing the use of the pentameric sequence of FWLHA as the On marker.

In summary, identification and/or analysis of *H. pylori* virulence factors is critical to the efforts to understand how *H. pylori* infects host cells and thus to combat gastric diseases caused by *H. pylori* infection. Our study not only validates the strong positive correlation between “on” status of the virulence factor, OipA and gastric cancer, but also elucidates a motif (FWLHA) in the signal peptide as an accurate determinant of OipA “On” status. Finally, the first structural analysis of OipA, indicates that it plays a central role in *H. pylori* pathogenesis as an autotransporter. As established by our comprehensive analysis, the significance of OipA as a virulence factor and prognostic marker for *H. pylori*-associated diseases underscores its potential as an effective therapeutic target.

### Electronic supplementary material

Below is the link to the electronic supplementary material.

Supplementary Material 1

Supplementary Material 2

Supplementary Material 3

## Data Availability

DNA sequences of the signal peptides collected from the patients were deposited into GenBank with the ID number 2,721,150. All the DNA and protein sequences of the signal peptides collected from the both patients and literature with different *oipA* states are available within Supplementary Information 1.
