# Supplementary figures and images for: Effect of the switch status of Helicobacter pylori outer inflammatory protein A on gastric diseases
Source: AMB Express. 2023 Oct 10;13:109. doi: 10.1186/s13568-023-01621-z (PMC10564699; doi:10.1186/s13568-023-01621-z)

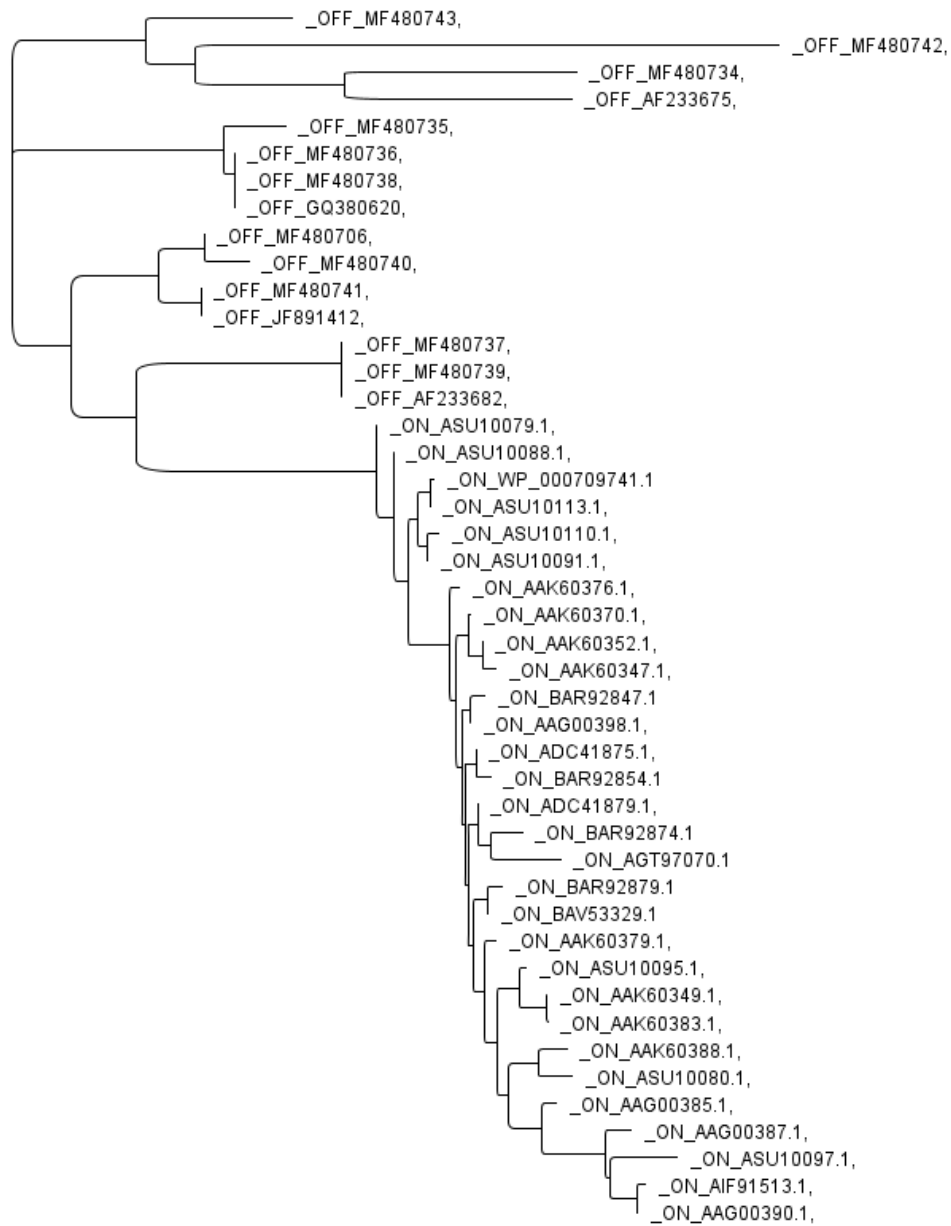

Phylogenetic tree of OipA signaling peptides that belong to “on” and off status.

Supplement: Supplementary file 1 — Supplementary Material 1 [file 13568_2023_1621_MOESM1_ESM.pdf]

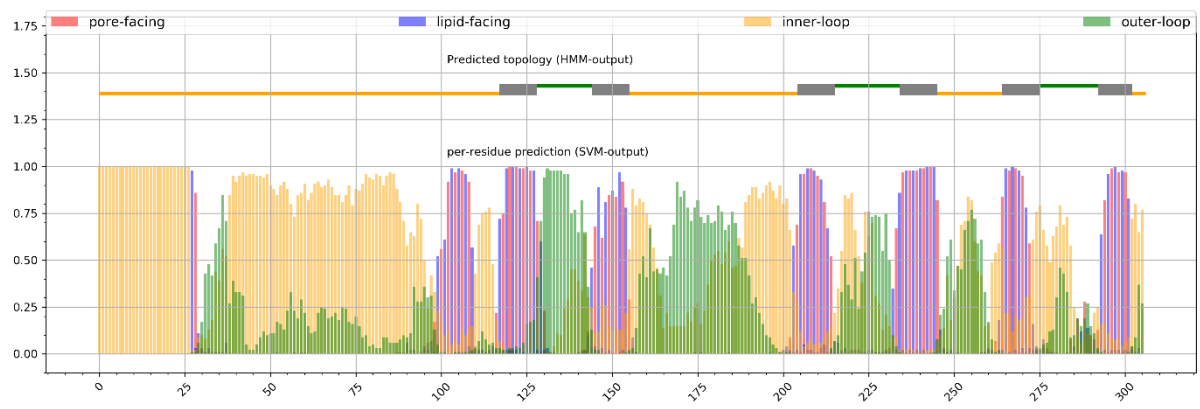

Figure 1 : OipA Topology Modeling

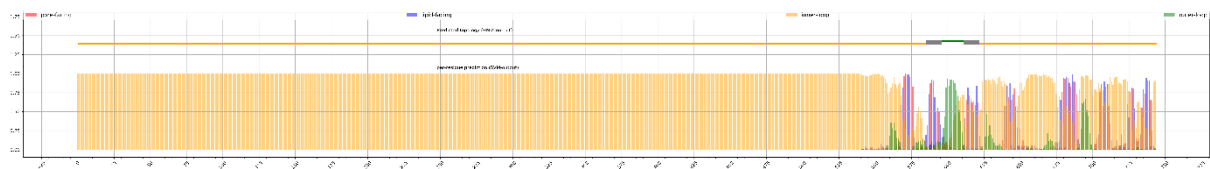

Figure 2: BabA Topology Modeling

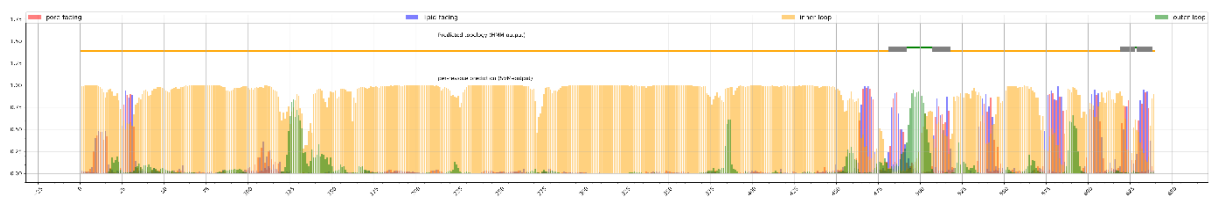

Figure 3: SabA Topology Modeling

Supplement: Supplementary file 2 — Supplementary Material 2 [file 13568_2023_1621_MOESM2_ESM.pdf]
