# Supplementary material for: Effect of the switch status of Helicobacter pylori outer inflammatory protein A on gastric diseases
Source: AMB Express. 2023 Oct 10;13:109. doi: 10.1186/s13568-023-01621-z (PMC10564699; doi:10.1186/s13568-023-01621-z)
Supplement: Supplementary file 3 — Supplementary Material 3 [file 13568_2023_1621_MOESM3_ESM.pdf]

Ref.numbe	Nucleotide Sequences	Number of CT Repeats	ON/OFF Status	Disease Information	Strain Information	Protein Sequences	Protein Sequences With Stop Codon (*)
[illegible]	[illegible]	[illegible]	[illegible]	[illegible]	[illegible]	[illegible]	[illegible]

4	ATG AAA AAA GCT CTC TTA CTC TCT CTC TCT CTC TCT CGT T		9 OFF	not specified	MKKALLLSLSLSR	MKKALLLSLSLSR*
4	ATG AAA AAA GCT CTC TTA CTC TCT CTC TCT CTC TCT CGT T		9 OFF	not specified	MKKALLLSLSLSR	MKKALLLSLSLSR*
4	ATG AAA AAA GCT CTC TTA CTC TCT CTC TCT CTC TCT CGT T		9 OFF	not specified	MKKALLLSLSLSR	MKKALLLSLSLSR*
4	ATG AAA AAA GCT CTC TTA CTA ACT CTC TCT CTC TCT CTC GTT		11 OFF	not specified	MKKALLLTLSLSLSV	MKKALLLTLSLSLSV*
5	ATG AAA AAA GCT CTC TTA CTA ACT CTC TCT CTC TCG TTC TGG CTC CAC GCT		6 ON	not specified	M K K A L L L T L S L S F W L H A	M K K A L L L T L S L S F W L H A
5	ATG AAA AAA GCT CTC TTA CTA ACT CTC TCT CTC TCT CGT TCT GGC TCC ACG		7 OFF	not specified	M K K A L L L T L S L S R S G S T	M K K A L L L T L S L S R S G S T*
5	ATG AAA AAA GCT CTC TTA CTA ACT CTC TCT CTC TCT CTC GTT CTG GCT CCA CGC		8 OFF	not specified	M K K A L L L T L S L S L V L A P R	M K K A L L L T L S L S L V L A P R*
6	ATG AAA AAA GCT CTC TTA CTA ACT CTC TCT CTC TTG TTT TGG CTC	(1+4)5	ON	chronic gastritis	M K K T L L L T F S L S F W L	M K K T L L L T F S L S F W L
6	ATG AAA AAA GCC CTC TTA CTA ACT CTC TCT CTC TCG TTT TGG CTC		6 ON	chronic gastritis	M K K A L L L T L S L S F W L	M K K A L L L T L S L S F W L
6	ATG AAA AAA GCC CTC TTA CTA ACT CTC TCT CTC TCT CTC GTT TTG GCT		8 OFF	chronic gastritis	M K K A L L L T L S L S L V L A	M K K A L L L T L S L S L V L A*
6	ATG AAA AAA GCT CTC TTA CTA ACT CTC TCT CTC TCG TTT TGG CTC		6 ON	gastric cancer	M K K A L L L T L S L S F W L	M K K A L L L T L S L S F W L
6	ATG AAA AAA GCT CTC TTA CTA ACT CTC TCT CTC TCG TTT TGG CTC		6 ON	gastric cancer	M K K A L L L T L S L S F W L	M K K A L L L T L S L S F W L
6	ATG AAA AAA GCT CTC TTA CTA ACT CTC TCT CTC TCG TTT TGG CTC		6 ON	gastric cancer	M K K A L L L T L S L S F W L	M K K A L L L T L S L S F W L
6	ATG AAA AAA GCT CTC TTA CTA ACT CTC TCT CTC TCG TTT TGG CTC		6 ON	gastric cancer	M K K A L L L T L S L S F W L	M K K A L L L T L S L S F W L
6	ATG AAA AAA GCT CTC TTA CTA ACT TTC TCT CTC TCG TTT TGG CTC	(1+4)5	ON	normal	M K K T L L L T F S L S F W L	M K K T L L L T F S L S F W L
6	ATG AAA AAA GCT CTT TTA CTC TCT CTC TCT CTC TCT CTC TCT CTCGTT TTT GGCT		10 OFF	normal	M K K A L L L S L S L S L S L V L A	M K K A L L L S L S L S L S L V L A*
7	ATG AAA AAG GCC CTC TTA CTA ACT CTC TCT CTC TCG TTT TGG CTC		6 ON	chronic gastritis	M K K A L L L T L S L S F W L	M K K A L L L T L S L S F W L
7	ATG AAA AAG GCC CTC TTA CTA ACT CTC TCT CTC TCG TTT TGG CTC		6 ON	chronic gastritis	M K K A L L L T L S L S F W L	M K K A L L L T L S L S F W L
7	ATG AAA AAG GCC CTC TTA CTA ACT CTC TCT CTC TCG TTT TGG CTC		6 ON	chronic gastritis	M K K A L L L T L S L S F W L	M K K A L L L T L S L S F W L
7	ATG AAA AAG GCC CTC TTA CTA ACT CTC TCT CTC TCG TTT TGG CTC		6 ON	chronic gastritis	M K K A L L L T L S L S F W L	M K K A L L L T L S L S F W L
[illegible]	[illegible]	[illegible]	[illegible]	[illegible]	[illegible]	[illegible]

10	ATG AAA AAA GCT CTC TTA CTA ACT TTC TCT CTC TCG TTT TGG CTC	(1+4)		ON	gastric cancer	M K K A L L L T F S L S F W L	M K K A L L L T F S L S F W L
10	ATG AAA AAA GCT CTC TTA CTA ACT TTC TCT CTC TCG TTT TGG CTC	(1+4)		ON	gastric cancer	M K K A L L L T F S L S F W L	M K K A L L L T F S L S F W L
10	ATG AAA AAA GCT CTC TTA CTA ACT TTC TCT CTC TCG TTT TGG CTC	(1+4)		ON	gastric cancer	M K K A L L L T F S L S F W L	M K K A L L L T F S L S F W L
10	ATG AAA AAA GCY CTC TTA CTA ACT CTC TCT CTC TCT CTC TGT TTT GGC TC		9	ON	gastric cancer	M K K A L L L T L S L S L S F W L	M K K A L L L T L S L S L S F W L
10	ATG AAA AAA GCY CTC TTA CTA ACT CTC TCT CTC TCT CGT TTT GGC		7	OFF	gastric cancer	M K K A L L L T L S L S R F G	M K K A L L L T L S L S R F G*
10	ATG AAA AAA GCY CTC TTA CTA ACT CTC TCT CTC TCT CGT TTT GGC		7	OFF	gastric cancer	M K K A L L L T L S L S R F G	M K K A L L L T L S L S R F G*
